# Isocyanate Modified GO Shape-Memory Polyurethane Composite

**DOI:** 10.3390/polym12010118

**Published:** 2020-01-05

**Authors:** Yuanchi Zhang, Jinlian Hu

**Affiliations:** Institute of Textiles and Clothing, Hong Kong Polytechnic University, Hung Hom, Hong Kong 999077, China; yuanc.zhang@connect.polyu.hk

**Keywords:** graphene oxide, shape-memory composite, mechanical and memory properties, bone repair

## Abstract

Shape-memory composites have benefits for minimally invasive surgery, but their wider applications for bone repair are hindered by conflicts between the mechanical and memory performances, especially at load-bearing locations. In this study, we fabricated a graphene oxide shape-memory polyurethane composite through the chemical combination of graphene oxide and isocyanate, in order to realize satisfactory mechanical and shape-memory effects. As desired, a modulus of ~339 MPa and a shape recovery ratio of 98% were achieved, respectively, in the composite. In addition, finite element analysis demonstrated that, after being implanted in a defective bone through a minimally invasive treatment, where the highest stress was distributed at the implant–bone interface, this composite could offer a generated force during the recovery process. Furthermore, we also discuss the origins of the improved mechanical and memory properties of the composites, which arise from increased net-points and the stable molecular structure inside. Therefore, with its superior structure and properties, we envision that this shape-memory composite can provide new insights toward the practical application of shape-memory polymers and composites in the field of bone repair.

## 1. Introduction

There has been an interest in the study of smart materials, which are always closely related to human life, for more than fifty years. Since the term “shape-memory” was first reported as a smart material in 1941, shape-memory materials, especially shape-memory polymers (SMPs), have been developed quickly [1,2]. SMPs are polymers that are able to remember, and then recover to, their permanent shape under certain external stimuli, such as heat [3], light [4], electricity [5], water [6], and solvents [7]. With the help of this special function, SMPs could be used for minimally invasive treatments, where a device made of SMPs could be programmed to a smaller size and then recovered to its original one according to the specified stimulus, such as the body temperature after being implanted [8]. SMPs generally possess two components: net-points (hard segments) and switches (soft segments), which are responsible for determining the permanent shape and fixing the temporary shape at temperatures below the transition temperature (*T* < *T*_trans_), respectively [1]. The hard segments are usually made of diisocyanate and diol, as well as chemical, physical, and interpenetrated or interlocked supramolecular complexes, while the soft segments mostly consist of polyols or polyesters [9,10]. Based on the architecture, SMPs can be easily designed with different features by selecting specific components. Consequently, their advantages, including low cost, high shape recoverability, good processing ability and excellent biocompatibility, have encouraged a large number of researchers to investigate the potential of using SMPs for biomedical applications such as biodegradable self-tightening sutures [11], heart scaffolds [12], and minimally invasive vascular devices [13]. Our group has also reported several applications in this field, including nerve conduit [14], artificial muscle [15], and orthoses [16]. However, the low mechanical properties and unsatisfactory stability of SMPs always hinder their development in terms of bone repair implants, especially in load-bearing locations [17]. For instance, the modulus of cancellous bone is usually more than 100 MPa, but the modulus of pure polyurethane (PU) falls in the range of ∼10−50 MPa [18,19].

One common approach to improving the mechanical performance of SMPs is to introduce nanofillers into the SMP matrix [20,21]. Graphene is a kind of new material, composed of sp^2^-bonded carbon atoms, which has excellent mechanical, optical, and conductive properties, as well as good biocompatibility because of its special structure [22,23]. Graphene oxide (GO) has the same structure of single planar sheets, and the oxygen-containing groups on the surface contribute to an easy decoration for modifying the polymer matrix. GO is expected to play an important part in significantly improving the mechanical properties of the composite because it has a superior modulus of 1 TPa and could form a newly stable structure, as well as forming an interaction between the nanofillers and the matrix [17,24,25,26]. However, despite the advantages in the SMP/GO composite, there are still concerns. Mechanical properties are always improved, but this involves sacrificing the shape-memory effects [27]. Meng et al. found that the shape recovery ratio of a composite with 7% carbon nanotube had a more than 30% decrease compared with pristine SMPs [27]. Previously, a type of modified GO SMP was prepared by our group through the simple physical blending method [17], where pristine shape-memory polyurethane (SMPU) was synthesized by 4,4′-methylenebis(phenyl isocyanate) (MDI) and 1,4-butanediol (BDO) as the net-point segment, together with polycaprolactone-diol (PCL-diol) as the switch segment. However, the Young’s modulus of the pristine SMPU and the composite was relatively low. We believe that these insufficient mechanical performances are due to limited hard segments and unstable net-point structures in the SMPU/GO composite.

Herein, to address the limitations of the mechanical and memory performances in SMP composites, we developed an isocyanate modified GO/SMPU (SMPU/iGO) composite. GO was modified with MDI through covalent bonding in order to chemically blend it within the SMPU matrix. It is expected that the mechanical and memory properties of the composite could be significantly enhanced by the chemically bonded GO, by forming a stable molecular structure; thus, the composite could be used for minimally invasive treatments in bone repair. The physical blending of the SMPU/GO composite was regarded as the control group for comparison. As a proof of concept, Fourier transform infrared (FTIR) spectroscopy, Raman spectroscopy, scanning electron microscopy (SEM), wide-angle X-ray diffraction (WAXD) and swelling tests were conducted to demonstrate the successful modifications of GO and the structures of the synthesized composites. In addition, differential scanning calorimetry (DSC), tensile tests, and bending tests were carried out to confirm the thermal and mechanical performances, as well as the shape-memory properties. Finally, finite element analysis (FEA) was performed to simulate the generated stress evolution that occurs during the recovery process on the interface between the SMP/iGO composite and cancellous bone in a minimally invasive bone-repairing model.

## 2. Materials and Methods

### 2.1. Materials

PCL-diol (Wn~550, CAPA2054) was obtained from Perstorp (Shanghai, China) Chemical Industry Co., LTD. 4,4-methylenebis (phenyl isocyanate) (MDI), 1,4-butanediol (BDO), and GO sheets were all obtained from Sigma-Aldrich (Shanghai, China). The PCL-diol and BDO were dried in advance under a vacuum at 100 °C for 24 h to remove the moisture. GO sheets were used as received.

### 2.2. Preparation of the SMP Composites

**Figure 1:**
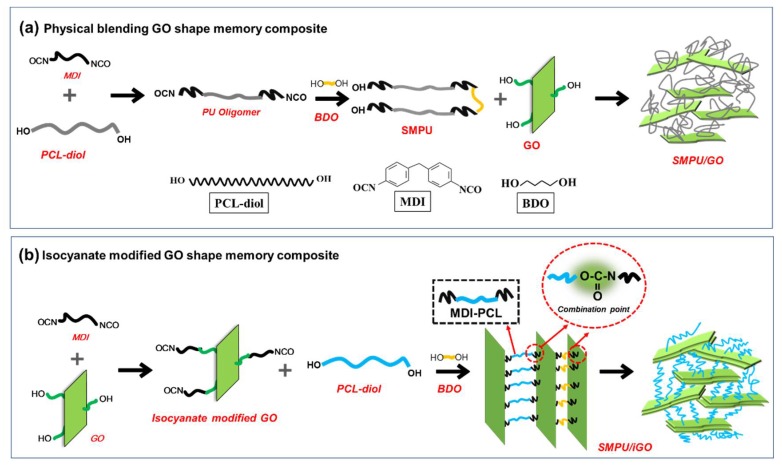
Preparation processes of composites: (**a**) shape-memory polyurethane (SMPU)/graphene oxide (GO) and (**b**) SMPU/isocyanate modified GO (iGO).

For the SMPU/GO composite, the SMPU was synthesized first through previously published procedures [8], and the steps can be found in Figure 1a. Regarding the SMPU/iGO composite, at first, GO was reacted with MDI at 80 °C for 4 h with mechanical stirring, where OH– and NCO– were combined, but leaving an excess of NCO–. Then, the PCL-diol was dispersed within iGO solutions at 80 °C for 3 h, and this was followed by adding the BDO with rapid mixing for about 40 s to extend the polymer chains. Finally, the product was poured into the mold to acquire the SMPU/iGO composite. The isocyanate and the polyol components were applied in a weight ratio so that the molar ratio of the NCO group and the OH group was fixed at 1.05. For both composites, the content of GO was 2 wt %, relative to the total composites. The process is shown in Figure 1b. The pristine SMPUs for both composites were also prepared in order to compare their mechanical and memory properties. As presented in Figure 1, we expect that the SMPU/iGO could have a more stable structure and an improved interaction between the nanofillers and the polymer chains.

### 2.3. Characterization of the SMPU Composites

A 2000 FTIR spectrometer (Perkin–Elmer, Suzhou, China) with attenuated total reflectance accessories was used in the wavenumber range of 650–4000 cm^-1^ in order to investigate the structure of the SMP/GO composites. Raman spectra were collected on a Bayspec spectrometer (San Jose, CA, USA) with a laser excitation wavelength of 785 nm, where the shift axis had been calibrated by silicon. SEM (FEI Inspect F50, Thermo Fisher, Hong Kong, China) at an accelerating voltage of 5 kV was utilized in order to recognize the surface morphologies of the composites. The wide-angle X-ray diffraction (WAXD) patterns of the samples were recorded using a Rigaku Smartlab XRD instrument (Oakland, CA, USA), using a Cu Ka radiation source (1.54 Å). The composites were scanned from 2θ = 10° to 80°. The swelling degree was tested as follows: (1) samples were dipped in dimethylacetamide (DMAC) at 60 °C for 24 h to achieve the equilibrium state, and the weights of the samples before and after dipping were recorded; (2) the samples were dried at 40 °C for 24 h, and the weights of the samples before and after drying were recorded. Then, the swelling degree was calculated according to the literature [28].

Glass transition temperatures (*T*_g_) were measured by using differential scanning calorimetry (DSC, Perkin Elmer DSC7, Shanghai, China). Samples were firstly heated to 120 °C and then cooled to 0 °C at a rate of 10 °C min^−1^. After remaining at this temperature for 5 min, a second scan was processed from 0 to 120 °C at the same rate, from which data were used for analyzing. Tensile tests of the SMP/GO composite were conducted at room temperature (RT) by using the INSTRON 5566 tester (Hong Kong, China) with an extension rate of 5 mm min^−1^. Young’s modulus, strain at break and stress were derived from the stress-strain curves to compare the mechanical properties. Shape-memory performance was investigated through a bending test and by recording the changes of the angles [8]. The original angle of the composite was noted as θ_0_, the maximum bending angle was θ_m_ at 80 °C, and the fixed angle of the sample at the equilibrium state was θ_f_. After being recovered at 80 °C, the final angle was θ_r_. Then, the shape fixity ratio (*R*_f_) and shape recovery ratio (*R*_r_) could be calculated according to the equations in the literature [8].

### 2.4. Finite Element Analysis

FEA was performed with the software Abaqus 6.14 (Dassault Systèmes, Vélizy-Villacoublay, France) to simulate the stress variety during the recovery process on the interface between the SMP/iGO composite and the bone tissues. We assumed that the device made of SMP/iGO was implanted into the defected bone at a compressed size, and we investigated the recovery stress at the interface when the temperature was 40 °C. The device–bone system was simplified to a cylinder-cube (materials and bone, respectively) model in this case, where all materials were regarded to be isotropic, homogeneous, and linearly elastic. According to the literature, the properties of the cylinder (materials) and the cube (bone) were set respectively as follows–Young’s modulus: 339 MPa/1.5 GPa, Poisson’s ratio: 0.25/0.3, CTE: 0.13511/0 [29,30].

### 2.5. Statistical Analysis

All data were statistically analyzed using the Origin 8.5 (OriginLab®, Northampton, MA, USA) and SPSS 22.0 software (IBM, Hong Kong, China). Moreover, results were compared using one-way ANOVA, and significant difference was set to *p* < 0.05.

## 3. Results and Discussion

### 3.1. Structures, Morphologies and Thermal Properties

**Figure 2:**
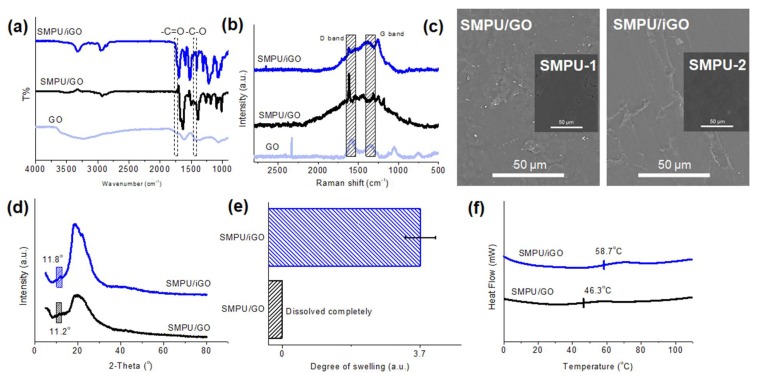
Characterization of the SMPU/iGO and SMPU/GO composites: (**a**) Fourier transform infrared (FTIR) spectra, in which the gray area indicates the bands at 1740 cm^−1^ and 1060 cm^−1^; (**b**) Raman spectra, in which the gray area indicates the D bands and G bands; (**c**) morphologies, in which the inserts are the SEM images of their own SMPU matrix—SMPU-1 for the SMPU/GO and SMPU-2 for the SMPU/iGO; (**d**) XRD results; (**e**) swelling degree; and (**f**) differential scanning calorimetry (DSC) results.

Figure 2 shows the characterization of the GO, SMPU and SMPU composites. At first, in the FTIR spectrum of GO (Figure 2a), the characteristic bands indicate several various types of oxygen functional groups: the broad band at 3204 cm^−1^ represents the groups of –OH, and the strong bands at 1740 cm^−1^ and 1060 cm^−1^ are the stretching vibration of the C=O carbonyl groups and the C–O groups, respectively [31]. In the spectra of the composites, these two characteristic bonds become stronger because the amount of corresponding carbon functional groups increases in the matrix, which can indicate the successful modifications of GO with SMPU. In the Raman spectra (Figure 2b), the G bands at 1345 cm^−1^ and the D bands at 1590cm^−1^ that arise from the sp^2^ and sp^3^ hybridizations of carbon, respectively, represent the existence of GO in the SMPU composites [32,33]. The intensity ratios of the D band and the G band (*I*_D_/*I*_G_), indicating the disorder degree, were calculated, and the values in the spectra of the SMPU/iGO and SMPU/GO composites were 0.82 and 1.32, respectively [34]. The lower I_D_/I_G_ ratio of the SMPU/iGO suggested a highly ordered structure in the composite [35,36], implying a more uniform dispersion of GO in the SMPU/iGO composites than the SMPU/GO composites. The morphologies of the composites are shown in Figure 2c, where the pristine SMPUs of both composites possess smooth surfaces. Compared with the SMPU/GO, the surface of SMPU/iGO had less roughness, illustrating the aggregation of the nanofillers in the SMPU/GO composite. The small peaks at 2θ = 11.8° and 11.2° in XRD patterns (Figure 2d) also confirm the existence of the GO nanofillers in the SMPU/iGO and SMPU/GO composites. Moreover, the interlayer spacing was calculated according to Bragg’s law, and the values were ~7.5 Å and ~7.9 Å in several cases. The smaller interlayer distance in SMPU/iGO suggests a compact and stable structure constituted by the nanofiller and the polymer chains. To investigate the crosslinked structures of the composites, swelling tests were conducted, and the results are shown in Figure 2e. The SMPU/iGO composite had a swelling degree of ~3.7, while the SMPU/GO could be dissolved completely. The differences suggested that GO combined with MDI through chemical bonds can successfully endow composites with a crosslinked network, which greatly improves their mechanical properties. Transition temperatures (*T*_g_) of the SMPU composites were investigated by DSC, as shown in Figure 2f, where the *T*_g_ of SMPU/GO and SMPU/iGO was around 46.3 and 58.7 °C, respectively. *T*_g_ could represent the mobility of the soft segment (polymer chains) in the matrix [36,37], and therefore the higher *T*_g_ in the SMPU/iGO suggested that the chemically bonded GO enhanced the whole structure and hindered the movement of the polymer chains in the amorphous phase, which could achieve greater strength improvement in the SMPU/iGO than in the SMPU/GO.

### 3.2. Mechanical Properties

**Figure 3:**
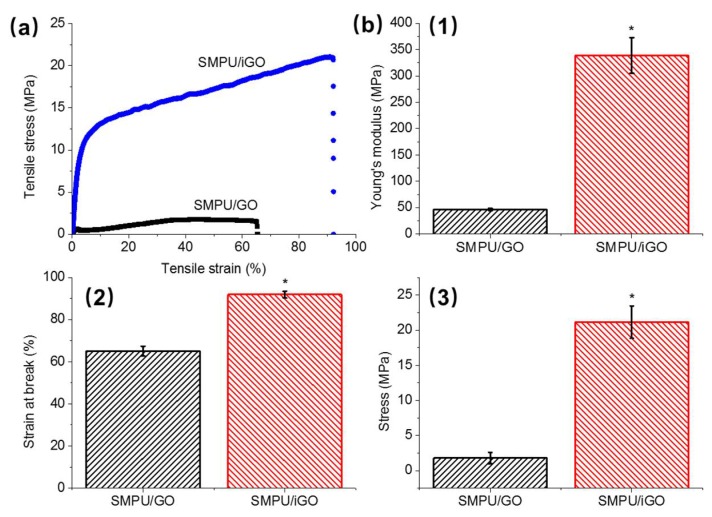
Mechanical properties of the SMPU/GO and SMPU/iGO: (**a**) tensile curves; (**b**) Young’s modulus, (**1**) strain at break, (**2**) and stress (**3**) derived from (**a**); *, significant difference compared to SMPU/GO, *p* < 0.05.

Figure 3a shows the tensile stress curve of the SMPU composites at RT. The tensile curves of the pristine SMPUs can be found in the Supplementary Information (Figure S1). Specifically, as shown in Figure 3(b1), SMPU/iGO has a modulus of ~339 MPa, which is significantly higher than that of the SMPU/GO (~47 MPa). Compared with their own pristine SMPU matrix, the SMPU/iGO had an improvement ratio of ~187%, while the SMPU/GO only had a ratio of ~74%. In addition, the strain at break and stress of the SMPU/iGO were, respectively, ~92% and ~21 MPa; however, the SMPU/GO had the values of ~65% and ~1.8 MPa (Figure 3(b2,b3)). These significant differences prove that the SMPU/iGO has robust mechanical properties compared with the physical blending composite. It also confirms our expectation based on the structural investigations and the DSC results, where the chemical incorporation of GO in the polymer matrix enhanced the net-points and formed a stable molecular construction.

### 3.3. Shape-Memory Effects

**Figure 4:**
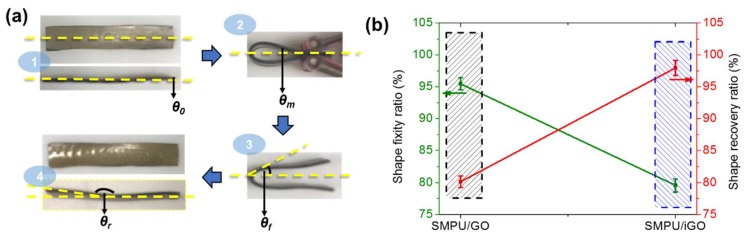
Shape-memory effects of SMPU/GO and SMPU/iGO: (**a**) Bending test—(**1**) original angle (θ_0_ = 0), (**2**) temporary angle (θ_m_ = 1800), (**3**) fixed angle (θ_r_), and (**4**) recovered angle (θ_r_); (**b**) Shape fixity ratios and recovery ratios.

The bending test was conducted in order to investigate the shape-memory properties. The procedures and the corresponding angles can be found in Figure 4a, and the calculated results can be found in Figure 4b. The shape-memory properties of the pristine SMPUs can be found in the Supplementary Information. As shown in Figure 4b, the SMPU/iGO had an *R*_f_ of ~80%, and the SMPU/GO had an *R*_f_ of ~95%, indicating that GO had more beneficial effects on the glassy state modulus of the physical blending composite rather than the chemically bonded one, since its shape was fixed in the glassy state. Nevertheless, the SMPU/iGO still had a satisfactory *R*_f_ to ensure good fixation ability for minimally invasive treatment. More importantly, the SMPU/iGO highly improved the *R*_r_ to ~98%. In general, the nanofillers could partly disturb the flexibility of the polymer in the elongated phase, meaning that the *R*_r_ would be lower, as with SMPU/GO (~80%), due to the aggregation of the nanofillers. However, in the structure of the SMPU/iGO, GO was regarded as the net-point in the new shape-memory system, resulting in a positive effect on the polymer’s elastic properties due to homogeneous dispersion. *R*_r_ is important in bone repair treatment, especially at load-bearing locations, because it corresponds to the recovery ability of the composites, the generated forces for providing the supporting function, and the mechanical stimulus to the surroundings in the defected site. The high *R*_r_ demonstrates that the SMPU/iGO has the potential for application in the field of bone repair.

### 3.4. FEA for the Recovery Process

**Figure 5:**
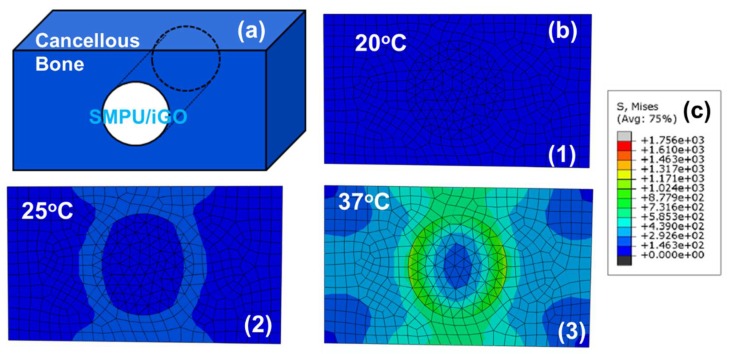
Finite element analysis (FEA) simulation of the SMPU/iGO: (**a**) the simplified material-bone model; (**b**) the equivalent stress distribution by FEA during the recovery process at 20, 25 and 37 °C; and (**c**) the scale of the stress distribution.

According to previous studies, a mechanical stimulus might improve cell and tissue growth [29,38,39]. Therefore, FEA was used to mimic the recovery process of the SMPU/iGO, after being implanted in the body through a minimally invasive surgery [29,40]. Figure 5a is a simplified model of the SMPU/iGO for bone repair, where the sample was compressed to a smaller size and then implanted in the defected bone. As temperature increased, the temporary shape tended to recover to its original state while being limited by the surrounding bone structure, resulting in increases in stress (Figure 5(b1,b2)). In the stress nephograms, the highest von Mises stresses could be found around the implant, particularly at the interface between the material and the bone. It was discovered that the generated stress was distributed from the center to the whole bone during heating. Figure 5c presents the scale of the stress, where the maximum value was around 1171 MPa at the interface. It was expected that the stress could be improved with increased temperature. The results suggest that the high von Mises stresses at the interface between the implant made of SMPU/iGO and the defect bone endow it with excellent mechanical and memory properties, meaning that the implant could be used for minimally invasive applications in high load-bearing bone repair.

### 3.5. Structural Models

**Figure 6:**
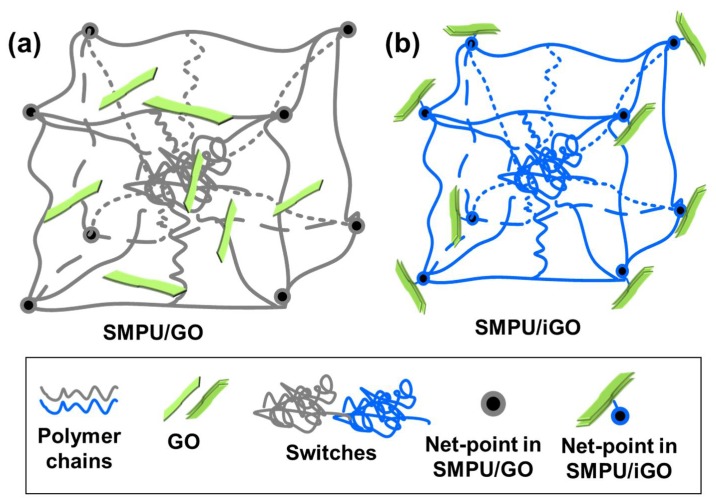
Structural models of the SMPU composites: (**a**) SMPU/GO, and (**b**) SMPU/iGO.

Here, we propose two models of modified GO SMP composites, based on the structural characterizations and the model we previously established for SMPs [1], in order to trace the origins of the improvements, as shown in Figure 6. At first, compared with SMPU/GO, SMPU/iGO has a more compact structure (Figure 6a,b), in which amorphous chains are regarded as switches in the two models. In the SMPU/GO, the net-points are short polymer chains composed of MDI and BDO, shown as the eight black points in the cube. GO nanofillers are dispersed randomly and sometimes aggregated, resulting in a higher *R*_f_ than that of the SMPU/iGO. As for the SMPU/iGO, GO is combined with MDI through covalent bonds. Consequently, the net-points are constituted by both GO and short polymer chains, as shown in Figure 6b. Moreover, the GO could be dispersed homogeneously, contributing to the enhancement of both the mechanical and memory properties of the composites.

## 4. Conclusions

This work proposes a chemically bonded SMPU composite using isocyanate modified GO for simultaneously improving the mechanical and memory properties. Physical blending SMPU/GO composites were used for comparison. The results demonstrated that the SMPU/iGO has a satisfactory modulus of 339 MPa (causing the range of the modulus of the cancellous bone to fall) and a stress of ~21 MPa with a good stretchability (~92%). More importantly for bone repair, SMPU/iGO has a beneficial shape-memory effect. In particular, the R_r_ of the SMPU/iGO was highly improved to ~98% compared with the physical blending composites. In addition to this, as expected, the SMPU/iGO presented a robust recovery process and generated force at the material–bone interface, as demonstrated by its behavior in the FEA. All in all, we believe that the developed SMPU/iGO with coalesced improved performances has the potential for practical application in the field of bone repair.

## Supplementary Materials

The following are available online at https://www.mdpi.com/2073-4360/12/1/118/s1, Figure S1: Mechanical (a) and memory properties (b) of the pristine SMPUs.

## References

[1] Hu J.L., Zhu Y., Huang H.H., Lu J. (2012). Recent advances in shape-memory polymers: Structure, mechanism, functionality, modeling and applications. Prog. Polym. Sci..

[2] Wang Z.L. (1998). Functional and Smart Materials.

[3] Hu J.L., Ji F.L., Wong Y.W. (2005). Dependency of the shape memory properties of a polyurethane upon thermomechanical cyclic conditions. Polym. Int..

[4] Lendlein A., Jiang H.Y., Junger O., Langer R. (2005). Light-induced shape-memory polymers. Nature.

[5] Meng Q.H., Hu J.L., Yeung L. (2007). An electro-active shape memory fibre by incorporating multi-walled carbon nanotubes. Smart Mater. Struct..

[6] Chen S.J., Hu J.L., Yuen C.W.M., Chan L.K. (2009). Novel moisture-sensitive shape memory polyurethanes containing pyridine moieties. Polymer.

[7] Du H.Y., Zhang J.H. (2010). Solvent induced shape recovery of shape memory polymer based on chemically cross-linked poly(vinyl alcohol). Soft Matter.

[8] Yuanchi Z., Jinlian H., Xin Z., Ruiqi X., Tingwu Q., Fenglong J. (2019). Mechanically robust shape memory polyurethane nanocomposites for minimally invasive bone repair. ACS Appl. Bio Mater..

[9] Zhang S., Yu Z., Govender T., Luo H., Li B. (2008). A novel supramolecular shape memory material based on partial α-cd–peg inclusion complex. Polymer.

[10] Zhu Y., Hu J.L., Luo H.S., Young R.J., Deng L.B., Zhang S., Fan Y., Ye G.D. (2012). Rapidly switchable water-sensitive shape-memory cellulose/elastomer nano-composites. Soft Matter.

[11] Lendlein A., Langer R. (2002). Biodegradable, elastic shape-memory polymers for potential biomedical applications. Science.

[12] Montgomery M., Ahadian S., Huyer L.D., Rito M.L., Civitarese R.A., Vanderlaan R.D., Wu J., Reis L.A., Momen A., Akbari S. (2017). Flexible shape-memory scaffold for minimally invasive delivery of functional tissues. Nat. Mater..

[13] Boire T.C., Gupta M.K., Zachman A.L., Lee S.H., Balikov D.A., Kim K., Bellan L.M., Sung H.J. (2015). Pendant allyl crosslinking as a tunable shape memory actuator for vascular applications. Acta Biomater..

[14] Chen C., Hu J.L., Huang H.H., Zhu Y., Qin T.W. (2016). Design of a smart nerve conduit based on a shape-memory polymer. Adv. Mater. Technol..

[15] Zhu S., Hu J. (2019). A titin inspired stress-memory polymer acts as a muscle. Mater. Chem. Front..

[16] Chen J., Hu J., Leung A.K., Chen C., Zhang J., Zhang Y., Zhu Y., Han J. (2018). Shape memory ankle-foot orthoses. ACS Appl. Mater. Interfaces.

[17] Xie R.Q., Hu J.L., Guo X., Ng F., Qin T.W. (2017). Topographical control of preosteoblast culture by shape memory foams. Adv. Eng. Mater..

[18] Montufar E., Casas-Luna M., Horynová M., Tkachenko S., Fohlerová Z., Diaz-de-la-Torre S., Dvořák K., Čelko L., Kaiser J. (2018). High strength, biodegradable and cytocompatible alpha tricalcium phosphate-iron composites for temporal reduction of bone fractures. Acta Biomater..

[19] Mano J.F., Sousa R.A., Boesel L.F., Neves N.M., Reis R.L. (2004). Bioinert, biodegradable and injectable polymeric matrix composites for hard tissue replacement: State of the art and recent developments. Compos. Sci. Technol..

[20] Xie R., Hu J., Ng F., Tan L., Qin T., Zhang M., Guo X. (2017). High performance shape memory foams with isocyanate-modified hydroxyapatite nanoparticles for minimally invasive bone regeneration. Ceram. Int..

[21] Feng X., Zhang G., Zhuo S., Jiang H., Shi J., Li F., Li H. (2016). Dual responsive shape memory polymer/clay nanocomposites. Compos. Sci. Technol..

[22] Li J., Cheng Y., Zhang S.Y., Li Y.J., Sun J., Qin C.X., Wang J.J., Dai L.X. (2017). Modification of go based on click reaction and its composite fibers with poly(vinyl alcohol). Compos. Part A Appl. Sci. Manuf..

[23] Cai Z.Q., Meng X.Y., Han Y.S., Ye H.M., Cui L.S., Zhou Q. (2015). Reinforcing polyamide 1212 with graphene oxide via a two-step melt compounding process. Compos. Part A Appl. Sci. Manuf..

[24] Pokharel P., Choi S., Lee D.S. (2015). The effect of hard segment length on the thermal and mechanical properties of polyurethane/graphene oxide nanocomposites. Compos. Part A Appl. Sci. Manuf..

[25] Thakur S., Karak N. (2014). Multi-stimuli responsive smart elastomeric hyperbranched polyurethane/reduced graphene oxide nanocomposites. J. Mater. Chem. A.

[26] Pasricha R., Gupta S., Srivastava A.K. (2009). A facile and novel synthesis of ag–graphene-based nanocomposites. Small.

[27] Meng Q., Hu J., Zhu Y. (2007). Shape-memory polyurethane/multiwalled carbon nanotube fibers. J. Appl. Polym. Sci..

[28] Shibata M., Ito T. (2003). Metallization of cross-linked polyurethane resins by reduction of polymer-incorporated metal ion. Polymer.

[29] Kitamura E., Stegaroiu R., Nomura S., Miyakawa O. (2004). Biomechanical aspects of marginal bone resorption around osseointegrated implants: considerations based on a three-dimensional finite element analysis. Clin. Oral Implants Res..

[30] Zhang Y., Hu J., Zhu S., Qin T., Ji F. (2019). A “trampoline” nanocomposite: uning the interlayer spacing in graphene oxide/polyurethane to achieve coalesced mechanical and memory. Compos. Sci. Technol..

[31] Wang R., Wang X., Chen S., Jiang G. (2012). In situ polymerization approach to poly(ε-caprolactone)-graphene oxide composites. Des. Monomers Polym..

[32] Yoo H.J., Mahapatra S.S., Cho J.W. (2014). High-speed actuation and mechanical properties of graphene-incorporated shape memory polyurethane nanofibers. J. Phys. Chem. C.

[33] Tan L., Gan L., Hu J.L., Zhu Y., Han J.P. (2015). Functional shape memory composite nanofibers with graphene oxide filler. Compos. Part. A Appl. S.

[34] Kudin K.N., Ozbas B., Schniepp H.C., Prud’Homme R.K., Aksay I.A., Car R. (2008). Raman spectra of graphite oxide and functionalized graphene sheets. Nano Lett..

[35] Tian K., Su Z., Wang H., Tian X., Huang W., Xiao C. (2017). N-doped reduced graphene oxide/waterborne polyurethane composites prepared by in situ chemical reduction of graphene oxide. Compos. Part A Appl. Sci. Manuf..

[36] Wu G., Xu X., He X., Yan Y. (2018). Preparation and characterization of graphene oxide-modified sapium sebiferum oil-based polyurethane composites with improved thermal and mechanical properties. Polymers.

[37] Tien Y.I., Wei K.H. (2002). The effect of nano-sized silicate layers from montmorillonite on glass transition, dynamic mechanical, and thermal degradation properties of segmented polyurethane. J. Appl. Polym. Sci..

[38] Powell C.A., Smiley B.L., Mills J., Vandenburgh H.H. (2002). Mechanical stimulation improves tissue-engineered human skeletal muscle. Am. J. Physiol. Cell Physiol..

[39] Fahy N., Alini M., Stoddart M.J. (2018). Mechanical stimulation of mesenchymal stem cells: Implications for cartilage tissue engineering. J. Orthop. Res..

[40] Wieding J., Souffrant R., Fritsche A., Mittelmeier W., Bader R. (2012). Finite element analysis of osteosynthesis screw fixation in the bone stock: an appropriate method for automatic screw modelling. PLoS ONE.

