# Supplementary material for: Isocyanate Modified GO Shape-Memory Polyurethane Composite
Source: Polymers (Basel). 2020 Jan 5;12(1):118. doi: 10.3390/polym12010118 (PMC7022938; doi:10.3390/polym12010118)
Supplement: Supplementary file 1 [file polymers-12-00118-s001.pdf]

polymers

MDPI

Article

Isocyanate Modified GO Shape-Memory Polyurethane Composite

Yuanchi Zhang [1], Jinlian Hu [1,*]

[1] Institute of Textiles and Clothing, Hong Kong polytechnic university, Hung Hom, Hong Kong, China; yuanc.zhang@connect.polyu.hk

* Correspondence: jin-lian.hu@polyu.edu.hk; Tel.: +852 2766 6437

Received: 6 November 2019; Accepted: 21 December 2019; Published: 5 January 2020

Supplementary Information:

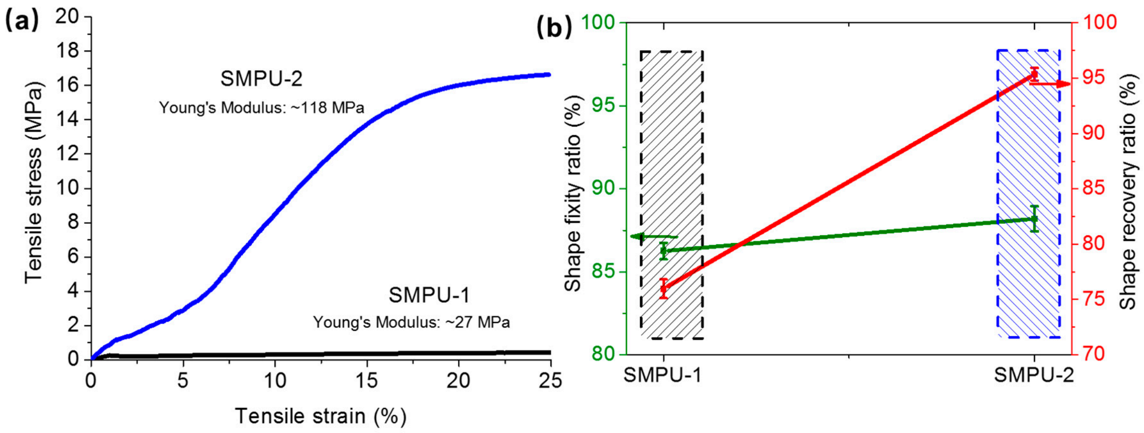

Figure S1. Mechanical (**a**) and memory properties (**b**) of the pristine SMPUs

© 2020 by the authors. Licensee MDPI, Basel, Switzerland. This article is an open access article distributed under the terms and conditions of the Creative Commons Attribution (CC BY) license (http://creativecommons.org/licenses/by/4.0/).
